# CORRELATIONS OF GPS-BASED COMMUNITY MOBILITY METRICS BETWEEN PERSONS WITH DEMENTIA AND FAMILY CAREGIVERS

**DOI:** 10.1093/geroni/igac059.1767

**Published:** 2022-12-20

**Authors:** Jane Chung, Joseph Boyle, David Wheeler

**Affiliations:** Virginia Commonwealth University, Richmond, Virginia, United States; Virginia Commonwealth University, Richmond, Virginia, United States; Virginia Commonwealth University, Richmond, Virginia, United States

## Abstract

Currently, the majority of dementia care is provided at home by informal caregivers. Most informal caregivers share a routine with their loved ones and change their activity patterns to adapt to a new routine of persons with dementia (PWDs). Given the dementia caregiving context, caregivers’ mobility behaviors and PWDs’ mobility may be positively associated. This study aimed to characterize patterns of GPS-derived community mobility in dementia dyads and examine relationships between PWDs’ and caregivers’ mobility patterns. Six dyads wore a GPS data logger inside and outside the home for 8-11 days. Twelve participants generated valid GPS track files (N=110). Four temporal and spatial mobility metrics were derived from GPS data (total distance, time use, median speed, and convex hull area). Then we calculated Pearson correlation coefficients between PWDs and their caregivers over all tracks. All dyads made active out-of-home trips, indicated by mean daily distance (range: 6,198 - 115,592m for PWDs; 5,125 - 108,857m for caregivers). Median speed of movement ranged from 0.09 to 1.29 km/hour for PWDs, and from 0.21 to 0.97 km/hour for caregivers. The mean size of convex hull over the monitoring period indicates a limited space usage level in both PWDs and caregivers, meaning restricted community mobility despite relatively large distance trips. The correlation coefficient was positive and significant for each metric (r = 0.70-0.97, p < .001). These results suggest substantial agreement in the mobility metrics between PWD and their caregivers, indicating a high level of dyadic effects of a partner’s experience of community mobility.

